# Pollen-mediated gene flow ensures connectivity among spatially discrete sub-populations of *Phalaenopsis pulcherrima*, a tropical food-deceptive orchid

**DOI:** 10.1186/s12870-019-2179-y

**Published:** 2019-12-30

**Authors:** Zhe Zhang, Stephan W. Gale, Ji-Hong Li, Gunter A. Fischer, Ming-Xun Ren, Xi-Qiang Song

**Affiliations:** 1 Key Laboratory of Genetics and Germplasm Innovation of Tropical Special Forest Trees and Ornamental Plants (Hainan University), Ministry of Education, College of Forestry, Hainan University, Haikou, People’s Republic of China; 2 Key Laboratory of Germplasm Resources of Tropical Special Ornamental Plants of Hainan Province, College of Forestry, Hainan University, Haikou, People’s Republic of China; 3 Kadoorie Farm & Botanic Garden, Lam Kam Road, Tai Po, Hong Kong, People’s Republic of China; 4 Center for Terrestrial Biodiversity of the South China Sea, Hainan University, Haikou, People’s Republic of China

**Keywords:** Fine-scale genetic structuring, Gene flow, Orchidaceae, Outcrossing mating system, Paternity analysis, Self-sterility

## Abstract

**Background:**

Gene flow in plants via pollen and seeds is asymmetrical at different geographic scales. Orchid seeds are adapted to long-distance wind dispersal but pollinium transfer is often influenced by pollinator behavior. We combined field studies with an analysis of genetic diversity among 155 physically mapped adults and 1105 F1 seedlings to evaluate the relative contribution of pollen and seed dispersal to overall gene flow among three sub-populations of the food-deceptive orchid *Phalaenopsis pulcherrima* on Hainan Island, China*.*

**Results:**

*Phalaenopsis pulcherrima* is self-sterile and predominantly outcrossing, resulting in high population-level genetic diversity, but plants are clumped and exhibit fine-scale genetic structuring. Even so, we detected low differentiation among sub-populations, with polynomial regression analysis suggesting gene flow via seed to be more restricted than that via pollen. Paternity analysis confirmed capsules of *P. pulcherrima* to each be sired by a single pollen donor, probably in part facilitated by post-pollination stigma obfuscation, with a mean pollen flow distance of 272.7 m. Despite limited sampling, we detected no loss of genetic diversity from one generation to the next.

**Conclusions:**

Outcrossing mediated by deceptive pollination and self-sterility promote high genetic diversity in *P. pulcherrima*. Long-range pollinia transfer ensures connectivity among sub-populations, offsetting the risk of genetic erosion at local scales.

## Background

Plants disperse genes via both pollen and seeds, but the contribution of either mode to total gene flow may be asymmetrical at different temporal and spatial scales [1, 2]. An outcrossing mating system and long-distance gene flow are likely to be critical for maintaining connectivity among populations and could potentially counteract the risk of genetic erosion associated with colonization bottlenecks, inbreeding and drift [3, 4]. In contrast, a selfing mating system with limited seed and pollen dispersal typically results in isolation-by-distance and lower genetic diversity within populations, leading to increased genetic differentiation among populations [5]. Clarifying patterns of gene dispersal within and between populations is key to developing an understanding of how genetic structure is shaped by these ecological processes [5–7].

Unlike most angiosperms, orchid pollen is aggregated into discrete pollen masses or pollinia that usually detach from the anther as a single unit together with accessory structures (such as a stipe, caudicle and viscidium) to facilitate transportation by pollinators. Consequently, orchid pollination is typically achieved in one effective visit, with only a single pollinator required to carry away all male gametes from a single flower and deliver them to the stigma of another [6, 8]. Pollen flow in orchids is therefore usually limited by pollinator behavior, with insect forage-range being closely linked to pollen dispersal distances [6, 9]. This appears to make pollen-mediated gene flow dominant at short to intermediate distances typically of less than one hundred meters [9, 10]. However, even if only one pollinium is successfully deposited onto a receptive stigma, the number of pollen grains it contains is sufficient to fertilize every ovule in the ovary [11]. As a result, all seeds in an orchid capsule are likely to be derived from a single pollen donor and therefore constitute full siblings [6, 12]. Nevertheless, studies using pollinia tracking methods demonstrate that although the stigma of most flowers in *Disa cooperi* [13] and *Satyrium longicauda* [14] accommodate pollinia from a single pollen donor, some receive pollinia from multiple candidate fathers. Further, Whitehead et al. (2015) [15] used microsatellite markers to reveal that multiple pollen donors can fertilize ovules in a single ovary in *Chiloglottis valida* and *C.* aff. *jeanesii*, providing the first genetic confirmation of polyandry in orchids.

Broadly speaking, orchids employ two distinct pollination systems, either rewarding or deceptive, each of which relies on different pollinator behaviors that in turn influence pollen flow distances and population genetic structure [8]. About one-third of all known orchid species are deceptive, achieving pollination without providing any floral reward [16]. Results from several studies suggest that deceptive pollination reduces geitonogamous pollination and promotes outcrossing, as deceived pollinators tend to travel further before alighting on another flower of the same orchid species, thereby enhancing pollen flow distances at local scales or among populations [17, 18]. As a result, deceptive orchids in particular exhibit lower genetic differentiation and higher pollen-mediated gene flow between local populations than do rewarding ones [19, 20]. However, deceptive pollination systems are usually associated with low levels of fruit set [21], which may result in low recruitment rates and reduced overall gene flow as compared to rewarding ones [22]. Whilst reduced overall gene flow may therefore restrict population growth, increased outcrossing and among-population pollen flow may help maintain genetic diversity and enhance inter-population connectivity [23].

Although predominantly outcrossing plants are generally expected to maintain high population genetic diversity [24], such species usually suffer more substantial losses of genetic variation when gene flow among populations is compromised following habitat fragmentation or decline [25, 26]. This is especially the case for insect-pollinated species [4], and the effect may be further compounded in orchids with small or spatially isolated populations, or for those with self-incompatible mating systems [27]. Although most orchids studied to date are self-compatible [28], some species have proved to possess self-pollination barriers [21, 29] and the vast majority of tropical species remain unexamined.

In contrast to pollen flow, the minute, wind-dispersed seeds of orchids are capable of traversing considerable distances [30]. Indeed, long-distance seed dispersal in orchids is known to promote the colonization of widely separated habitats, sometimes over hundreds of kilometers [30], as in the terrestrial orchid *Liparis loeselii* in which seed dispersal distances exceeding 220 km have been documented [31]. However, wind-tunnel experiments [32] and seed traps [33] suggest that most orchid seeds disperse rather more locally, with genetic studies demonstrating that most recruitment occurs close to mother plants [34–36]. Where this coincides with limited pollen flow, genetic structuring within populations can become more entrenched [37]. Nevertheless, even rare long-distance seed dispersal can reduce genetic structuring within populations and genetic differentiation among populations over larger spatial scales [38]. Correspondingly, seed flow typically exhibits a highly leptokurtic distribution, with high values over shorter distances and a long, flat tail over greater distances [39]. As a result, orchids tend to exhibit relatively low levels of genetic differentiation, even among widely disjunct populations [36], as compared with other plant families.

In the present study, we combine field studies with an analysis of gene flow among spatially discrete sub-populations of the food-deceptive orchid *Phalaenopsis pulcherrima* on Hainan Island, China. We sought to address the following questions: (i) How does *P. pulcherrima* ensure outcrossing in spatially isolated populations? (ii) Over what geographic scale is the species capable of achieving pollen flow? (iii) Is there evidence of fine scale genetic structuring, among-population genetic partitioning or an inter-generational bottleneck which might indicate that either pollen- or seed-mediated gene flow is insufficient to maintain large effective population size, population connectivity and genetic diversity?

## Results

### Floral development and breeding system

**Fig. 1:**
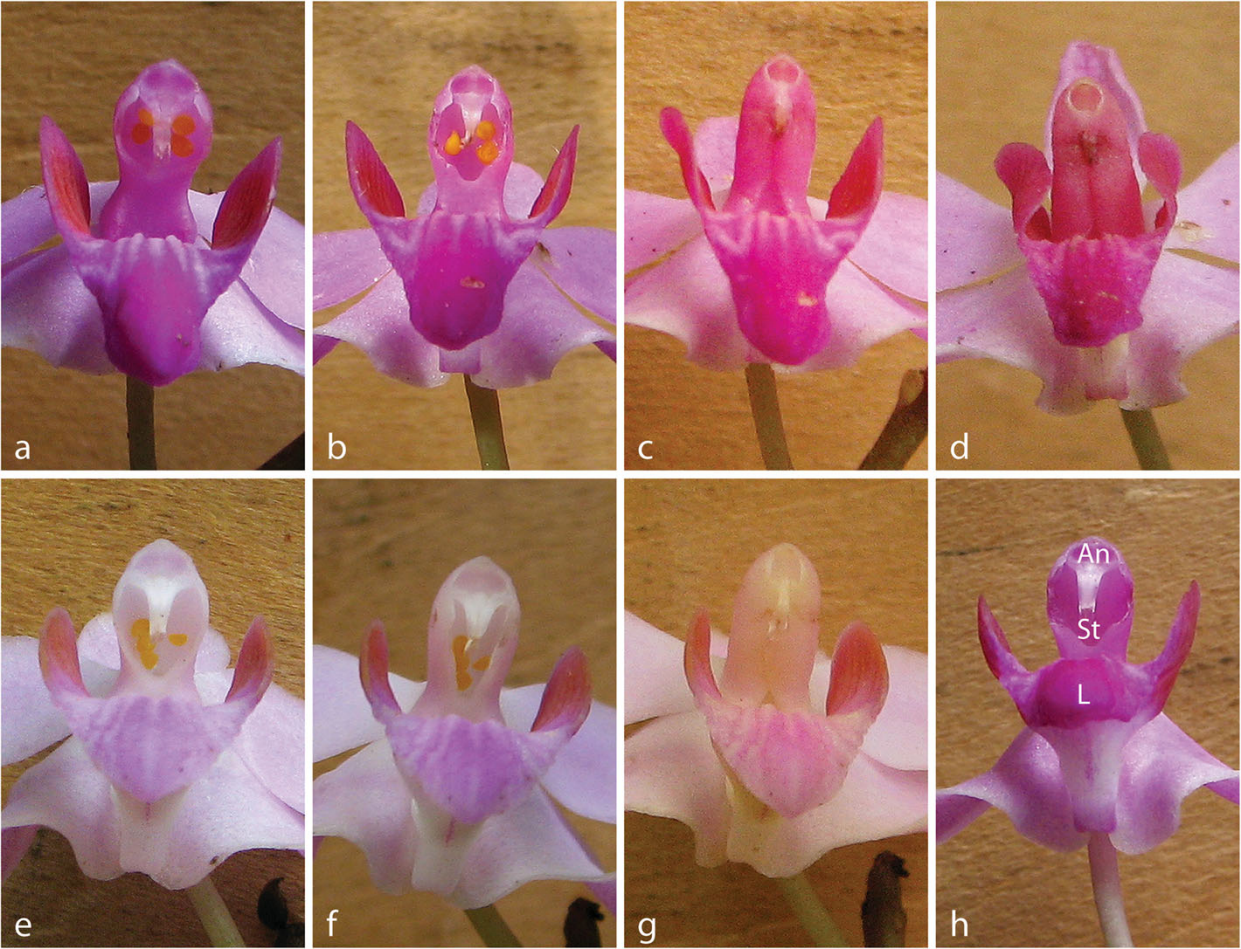
Stigma development after pollination treatment in *Phalaenopsis pulcherrima.* Self-pollination + 2 h, stigma still fully open (**a**), self-pollination + 9 h, rostellum beginning to enlarge (**b**), self-pollination + 24 h, rostellum almost fully obscuring the stigma (**c**), self-pollination + 48 h, stigma fully obscured (**d**), cross-pollination + 2 h, stigma still fully open (**e**), cross-pollination + 10 h, rostellum enlarged and partially obscuring the stigma (**f**), cross-pollination + 24 h, stigma fully obscured (**g**), control flower (no treatment) (**h**): An, Anther, St, Stigma, L, Labellum

The lifespan of a single flower without manipulation (*M* ± *SD*, 4.6 ± 2.5 days, *n* = 10) was significantly longer than that of an emasculated flower (2.8 ± 1.0 days, *n* = 10; *p* < 0.01). Following both artificial (self- and cross-) pollination treatments, the rostellum gradually enlarged, coalesced with and eventually obscured the stigma, but complete closure of the stigma occurred significantly more quickly following cross-pollination (8.2 ± 2.1 h, *n* = 10) than self-pollination (10.0 ± 2.1 h, *n* = 10; *p* < 0.05; Fig. 1).

Bagged and emasculated flowers failed to develop into fruit. Natural fruit-set as a result of open pollination (3.8%) was significantly lower than that for hand-pollination (*p* < 0.01), but there was no difference between the fruit-set of artificial self- (89.2%) and cross-pollinations (90.4%) (Additional file 1: Table S1).

**Fig. 2:**
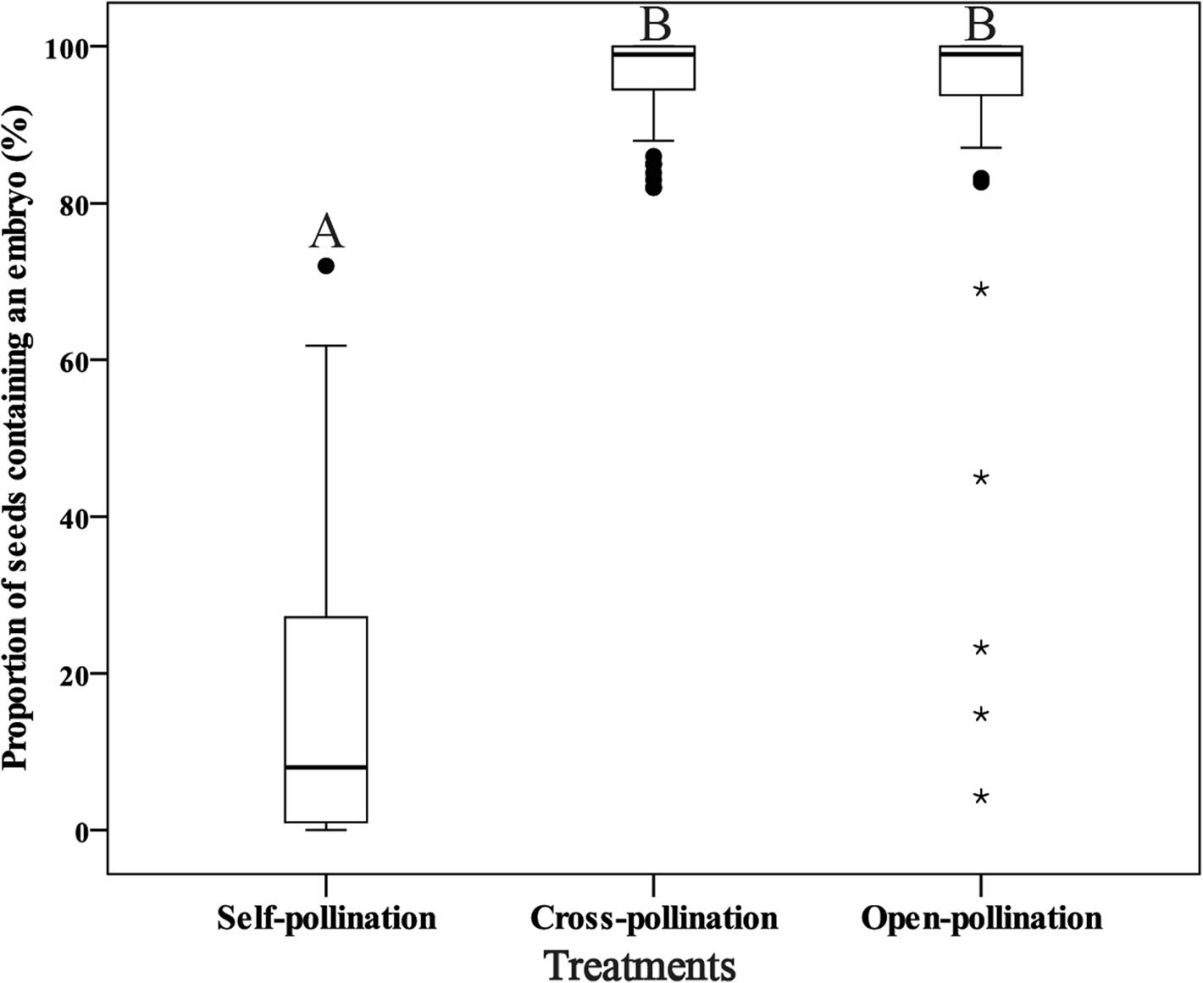
Seed set in artificially self- and cross-pollinated and open pollinated capsules of *Phalaenopsis pulcherrima*. Capital letters (A and B) indicate significance between treatments at 99% confidence interval. Asterisks indicate capsules with a seed set significantly lower than the mean

Seed-set was significantly lower in self-pollinated capsules (*M* ± *SD*, 16.1 ± 17.8%, range from 0.0–72.0%) than in cross-pollinated capsules (96.2 ± 5.0%, range from 82.0–100.0%) and open-pollinated capsules (93.7 ± 4.4%; Fig. 2). In addition, 11 (13.3%) of the self-pollinated capsules were empty and a further 55 (66.3%) had an extremely depressed seed set (< 20.0%). Only five of the 124 open-pollinated capsules had a seed set significantly lower than the mean (marked as asterisks in Fig. 2).

### Epifluorescence microscopy

Pollen grains on both self- and cross-pollinated stigmas did not germinate within the first two days (Additional file 2: Figure S1a, b). Pollen tubes were first observed three days after pollination, but the pollen grain germination frequency and pollen tube growth rate in the cross-pollination treatment far exceeded those in the self-pollination treatment (Additional file 2: Figure S1c, d). After four days, pollen tubes in the cross-pollination treatment had grown > 500 μm to the base of the style, whereas those in the self-pollination treatment had penetrated < 200 μm (Additional file 2: Figure S1e, f). After five days, masses of pollen tubes in the cross-pollination treatment had grown into the ovary and fertilized the ovules, whereas most pollen tubes in the self-pollination treatment had stalled midway along the column with only a few having proceeded as far as the ovary and very few having fertilized the ovules (Additional file 2: Figure S1 g, h).

### Genotypic diversity among adults and offspring

No null alleles were detected among all 15 microsatellite loci and high genetic diversity was confirmed. The average number of alleles per locus was 6.8 for adults and 5.0 for F1 seedlings (Table 1). The mean *H*_*o*_ and *H*_*e*_ were 0.656 and 0.656 among the adults, and 0.580 and 0.606 among the seedlings, respectively (Table 1). Although the calculated genetic diversity measures in the adults were higher than in the seedlings, the differences were not significant: *N*_*a*_ (*F* = 3.901, *p =* 0.058), *N*_*e*_ (*F* = 0.854, *p =* 0.363), *I* (*F* = 1.164, *p =* 0.290), *H*_*o*_ (*F* = 1.240, *p =* 0.275) and *H*_*e*_ (*F* = 0.564, *p =* 0.459). Heterozygote excess and significant deviation from HWE were detected in both adults and offspring (*p* < 0.05; Table 1). Overall *F*_*is*_ was − 0.014 for the adults and 0.031 for the offspring, but there was no significant difference between them (*F* = 0.615, *p =* 0.440). In addition, *F*_*is*_ in both adults and seedlings did not significantly differ from 0. Across all 15 loci, the cumulative expected exclusion probability of the first Pr(*Ex1*) and second Pr(*Ex2*) parents estimated from adults were 0.9950 and 0.9999, respectively (Table 1).

**Table 1:** Genetic diversity across 15 microsatellite loci within all adults (*n* = 155) and offspring (*n* = 1105). Number of observed alleles(*N*_*a*_), number of effective alleles(*N*_*e*_), Shannon’s information index (*I*), and, observed (*H*_*o*_) and expected (*H*_*e*_) heterozygosity, inbreeding coefficient (*F*_*is*_), and Pr (*Ex2*), paternity exclusion probability of the first [Pr (*Ex1*)] and second [Pr (*Ex2*)] parent. Highly significant departure from Hardy–Weinberg equilibrium (HWE) are indicated with asterisks (^***^p < 0.001, ^**^ *p* < 0.01, ^*^ *p* < 0.05)

	Adults (*n* = 155)								Offspring (*n* = 1105)					
Locus	*N*_*a*_	*N*_*e*_	*I*	*H*_*o*_	*H*_*e*_	*F*_*is*_	Pr (*Ex1*)	Pr (*Ex2*)	*N*_*a*_	*N*_*e*_	*I*	*H*_*o*_	*H*_*e*_	*F*_*is*_
L3	8	3.438	1.468	0.665	0.709	0.063^***^	0.696	0.518	5	2.944	1.328	0.603	0.660	0.084^***^
L6	4	2.319	0.977	0.677	0.569	−0.191^**^	0.838	0.692	4	2.825	1.068	0.693	0.646	−0.065^***^
L9	5	3.478	1.340	0.690	0.713	0.031^***^	0.709	0.537	3	2.926	1.085	0.532	0.658	0.193^***^
L22	7	2.968	1.285	0.729	0.663	−0.099^***^	0.751	0.591	4	2.562	1.074	0.269	0.610	0.561^***^
L29	9	4.202	1.653	0.742	0.762	0.026	0.627	0.447	6	3.824	1.374	0.800	0.739	−0.083^***^
L33	14	6.181	2.027	0.794	0.838	0.053^***^	0.487	0.319	8	4.927	1.740	0.800	0.797	−0.001^***^
L46	8	4.537	1.719	0.735	0.780	0.057	0.595	0.415	6	4.335	1.601	0.720	0.769	0.066^***^
L52	5	2.330	1.123	0.619	0.571	−0.085	0.818	0.647	5	2.130	1.024	0.566	0.530	−0.065^***^
L54	7	4.242	1.586	0.761	0.764	0.004^*^	0.628	0.447	6	3.937	1.489	0.764	0.746	−0.024^***^
L56	4	2.179	0.917	0.490	0.541	0.094	0.850	0.719	3	1.542	0.613	0.433	0.351	−0.233^***^
L53	2	1.290	0.385	0.258	0.225	−0.148	0.974	0.899	2	1.085	0.170	0.082	0.078	−0.042
L57	8	2.835	1.399	0.665	0.647	−0.027^***^	0.745	0.563	6	2.929	1.384	0.759	0.659	−0.150^***^
L64	11	4.902	1.880	0.787	0.796	0.011	0.552	0.373	8	4.625	1.715	0.855	0.784	−0.088^***^
L51	4	1.835	0.811	0.497	0.455	−0.092^***^	0.896	0.767	4	1.573	0.718	0.380	0.364	−0.040^***^
L31	6	5.058	1.685	0.729	0.802	0.091^***^	0.571	0.393	5	3.142	1.263	0.445	0.682	0.346^***^
Mean	6.8	3.453	1.350	0.656	0.656	−0.014^***^			5.0	3.025	1.178	0.580	0.606	0.031^***^
Total	102						0.9950	0.9999	80					

### Genotypic diversity and differentiation among sub-populations

For the three sub-populations, the average number of observed alleles varied from 5.333 at YJM-B to 6.133 at YJM-C (mean = 5.711; Table 2). *H*_*o*_ ranged from 0.646 at YJM-A to 0.668 at YJM-B (mean = 0.659), and *H*_*e*_ ranged from 0.638 at YJM-A to 0.659 at YJM-B (mean = 0.651; Table 2). There was no difference across the 15 loci among sub-populations for all calculated genetic diversity measures: *N*_*a*_ (*F* = 0.400, *p* = 0.673), *N*_*e*_ (*F* = 0.054, *p =* 0.948), *I* (*F* = 0.006, *p =* 0.994), *H*_*o*_ (*F* = 0.079, *p =* 0.924) and *H*_*e*_ (*F* = 0.072, *p =* 0.931). *F*_*is*_ was − 0.024, − 0.027 and − 0.024 for YJM-A, YJM-B and YJM-C, respectively (*F* = 0.002, *p =* 0.998). AMOVA revealed most genetic variation to lie within sub-populations (99.3%), as opposed to among sub-populations (0.7%; Table 3). The *F*_*st*_ value was very low but indicated significant differentiation between sub-populations (*p* < 0.01; Table 3).

**Table 2:** Genetic diversity in three sub-populations of *Phalaenopsis pulcherrima*. Number of individuals (*N*), number of observed alleles (*N*_*a*_), number of effective alleles (*N*_*e*_), Shannon’s information index (*I*), and, observed (*H*_*o*_) and expected (*H*_*e*_) heterozygosity, inbreeding coefficient (*F*_*is*_)

Sub-population	*N*	*N*_*a*_	*N*_*e*_	*I*	*H*_*o*_	*H*_*e*_	*F*_*is*_
YJM-A	56	6.133	3.470	1.319	0.646	0.638	−0.024
YJM-B	39	5.333	3.431	1.328	0.668	0.659	−0.027
YJM-C	60	5.667	3.310	1.310	0.662	0.655	−0.024
Total	155	5.711	3.404	1.319	0.659	0.651	−0.025

**Table 3:** Analysis of molecular variance (AMOVA) for the three sub-populations of *Phalaenopsis pulcherrima*. Sum of squares (*SS*), degree of freedom (*df*), F-statistics (*F*_*st*_). Significant difference is indicated with asterisk (^*^ *p* < 0.01)

Source	*SS*	*df*	Percentage	*F*_*st*_
Among sub-populations	16.534	2	0.007	0.007^*^
Within sub-population	1510.740	307	0.993	
Total	1527.274	309		

### Spatial distribution of individuals

**Fig. 3:**
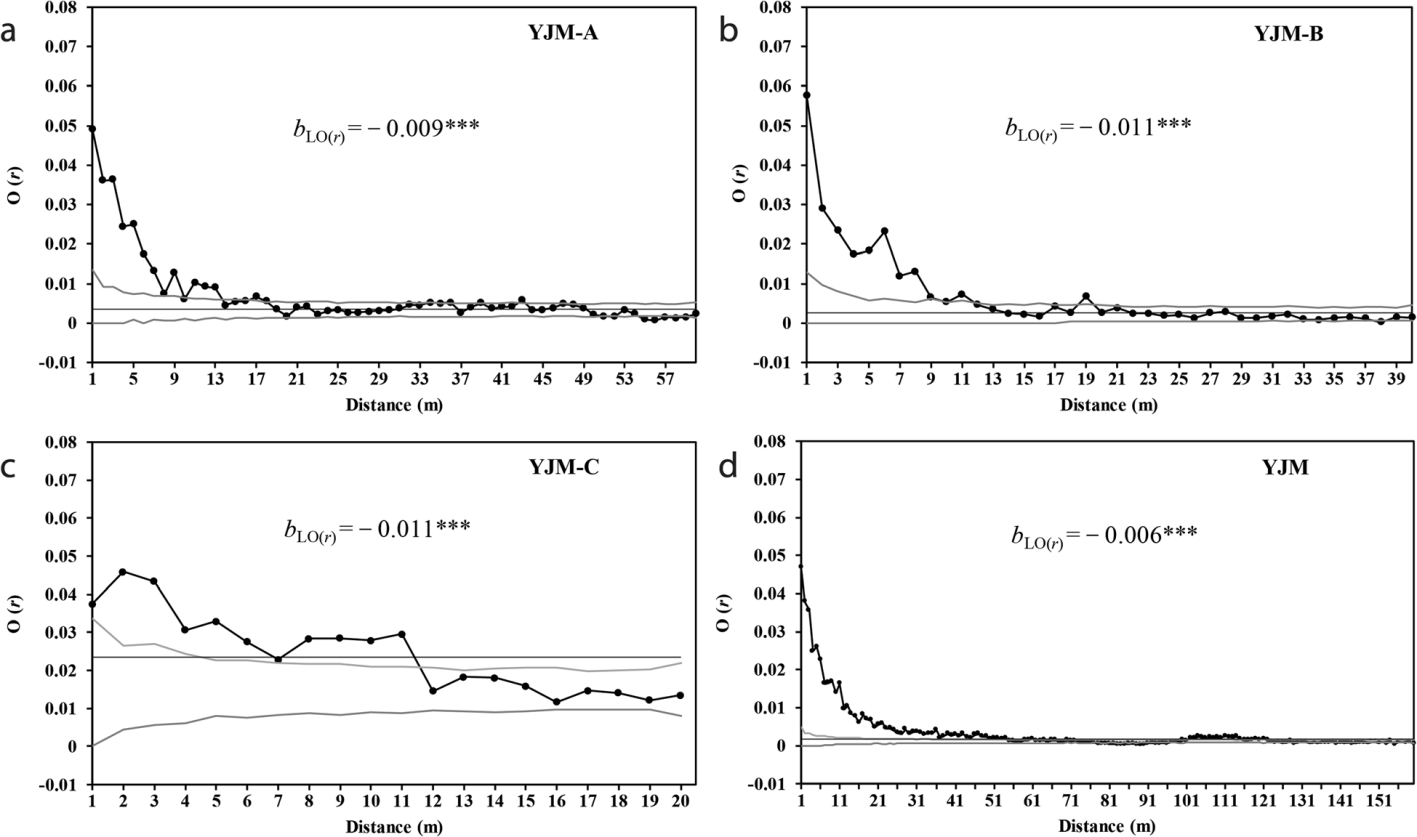
Observed O(*r*)-statistic estimates for univariate analysis of individuals of *Phalaenopsis pulcherrima* at each of the three sub-populations **a** YJM-A, **b** YJM-B, **c** YJM-C and **d** the whole population YJM. *b*_LO(*r*)_ represents the slope of the linear regression of the O-ring statistic, O(*r*) against log spatial distance, ln(*r*). Envelopes defined by the 5% highest and lowest values generated from 999 Monte Carlo simulations under the null hypothesis of complete spatial randomness (CSR) are indicated by grey lines. The thin solid line indicates the first-order intensity (λ) of the point pattern within (sub-)populations: 0.0035 in YJM-A, 0.0027 in YJM-B, 0.0160 in YJM-C and 0.0018 for YJM as a whole. ***p < 0.001

O-ring analysis demonstrated significant spatial aggregation of individuals (Fig. 3). This aggregation occurred at scales of *r* = 1–9, 11–13, 17–18, 36, 39 and 43 m at YJM-A; *r* = 1–11 and 19 m at YJM-B; *r* = 1–11 m at YJM-C; and *r* = 1–54, 61, 102–115 and 118–121 m for YJM as a whole (Fig. 3). However, the higher absolute value of O(*r*) occurred commonly at relatively fine scales (e.g. the value of O(*r*) exceeded 0.01 at distance classes of 1–7, 9 and 11–13 m at YJM-A). Weak but significant regular distribution of individuals also occurred at scales of *r* = 51, 52 and 55–59 m at YJM-A and at 126, 127, 129, 133–135, 138, 140, 142–145, 147, 148, 151, 153, 155, 158 and 160 m for YJM as a whole.

The O(*r*) function of ln(*r*) indicated a significant negative relationship (*p* < 0.001), with *b*_LO(*r*) =_ − 0.009 (95% CI: − 0.010, − 0.008) at YJM-A, − 0.011 (95% CI: − 0.013, − 0.009) at YJM-B, − 0.011 (95% CI: − 0.014, − 0.008) at YJM-C and − 0.006 (95% CI: − 0.007, − 0.005) for YJM as a whole (Fig. 3). All *b*_LO(*r*)_ values for each sub-population and for YJM as a whole were significantly lower than 0. However, *b*_LO(*r*)_ estimates for the three sub-populations were not significantly different from each other.

### Fine-scale genetic structure and dispersal estimates

**Fig. 4:**
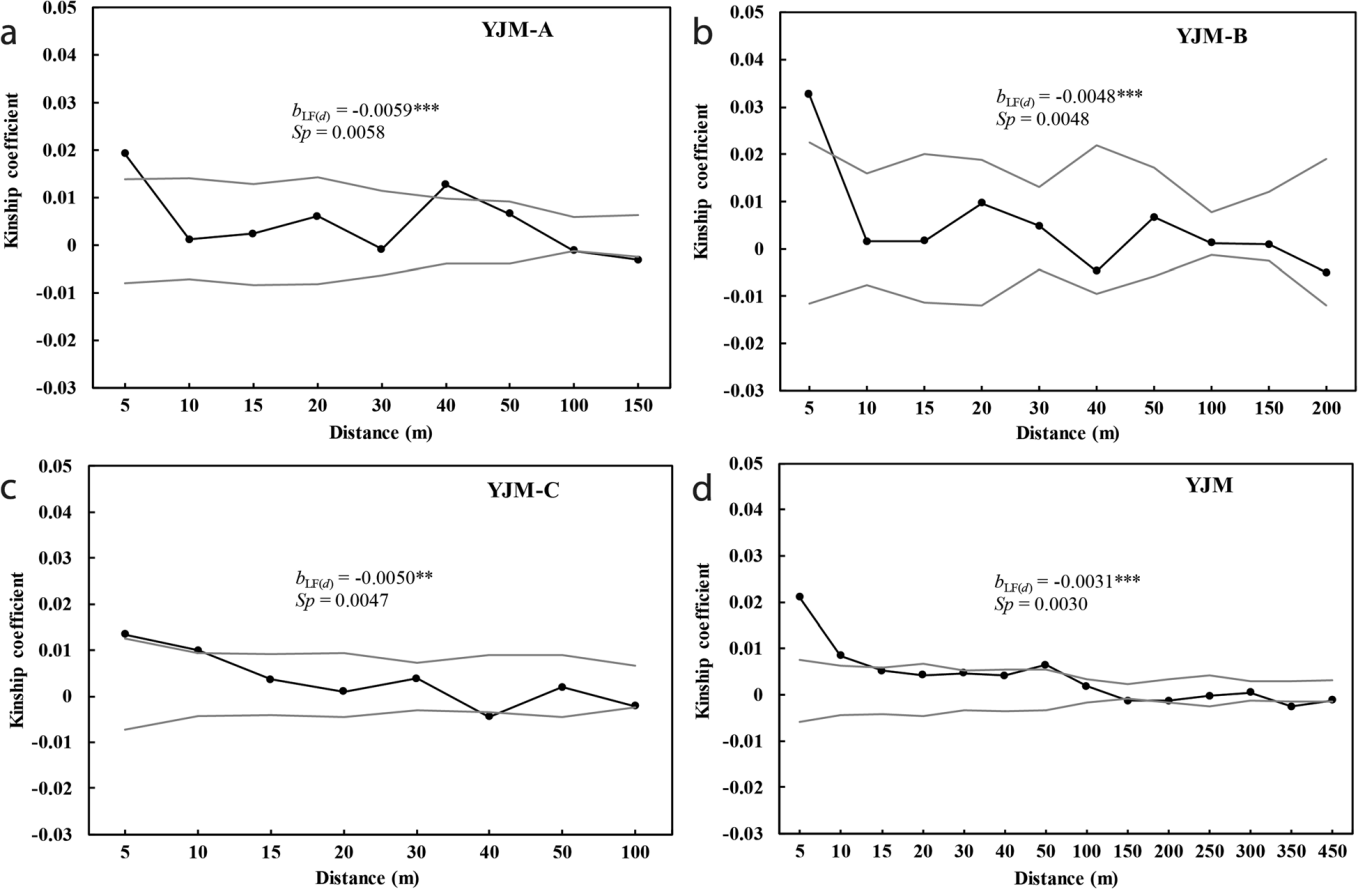
Correlograms of kinship coefficients (*F*_*ij*_) for adult individuals from sub-population **a** YJM-A, **b** YJM-B, **c** YJM-C and **d** the whole population YJM. Closed circles indicate mean coancestry values for each given distance classes. The dashed lines represent upper and lower 95% confidence envelopes around the null hypothesis of no genetic structure [*F*_*ij*_ (*d*) = 0]. *b*_LF(*d*)_ represents the slope of the regression of kinship coefficient values, F_*ij*_ (*d*), against log distance interval (*d*). **p < 0.01, ***p < 0.001

Fine-scale genetic structure analysis for the three sub-populations indicated positive and significant kinship at short distance intervals, with an *F*_*ij*_ of 0.0192 at 5 m at YJM-A, 0.0326 at 5 m at YJM-B, 0.0132 at 5 m at YJM-C, and 0.0210 at 5 m and 0.0085 at 10 m in the population as a whole (Fig. 4). At larger distance intervals, a significantly positive *F*_*ij*_ value was retrieved only at 40 m at YJM-A (0.0127) and at 50 m at YJM as a whole (0.0065). In contrast, significantly negative kinship coefficient values were observed at 40 m at YJM-C (− 0.0045), and at 150 m and 350 m for the population as a whole (− 0.0013 and − 0.0026 respectively).

The slope of the linear regression between F_*ij*_(*d*) and log geographic distances was significantly negative in all cases, with *b*_LF(*d*)_ = − 0.0059 (*p* < 0.001; 95% CI: − 0.0028, − 0.0089) for YJM-A, − 0.0048 (*p* < 0.001; 95% CI: − 0.0002, − 0.0093) for YJM-B, − 0.0050 (*p* < 0.01; 95% CI: − 0.0016, − 0.0084) for YJM-C, and − 0.0031 (*p* < 0.01; 95% CI: − 0.0020, − 0.0042) for the population as a whole (Fig. 4). However, the correlation did not differ significantly among the three sub-populations. The *Sp* statistic suggested that fine-scale genetic structure intensity was greatest at YJM-A with *Sp* = 0.0057 (95% CI: 0.0027, 0.0088), followed by YJM-B (0.0048; 95% CI: 0.0003, 0.0092) and YJM-C (0.0047; 95% CI: 0.0013, 0.0080); however, there were no significant differences among the three sub-populations. The lowest *Sp* values were observed in the population as a whole (0.0030; 95% CI: 0.0020, 0.0042).

**Fig. 5:**
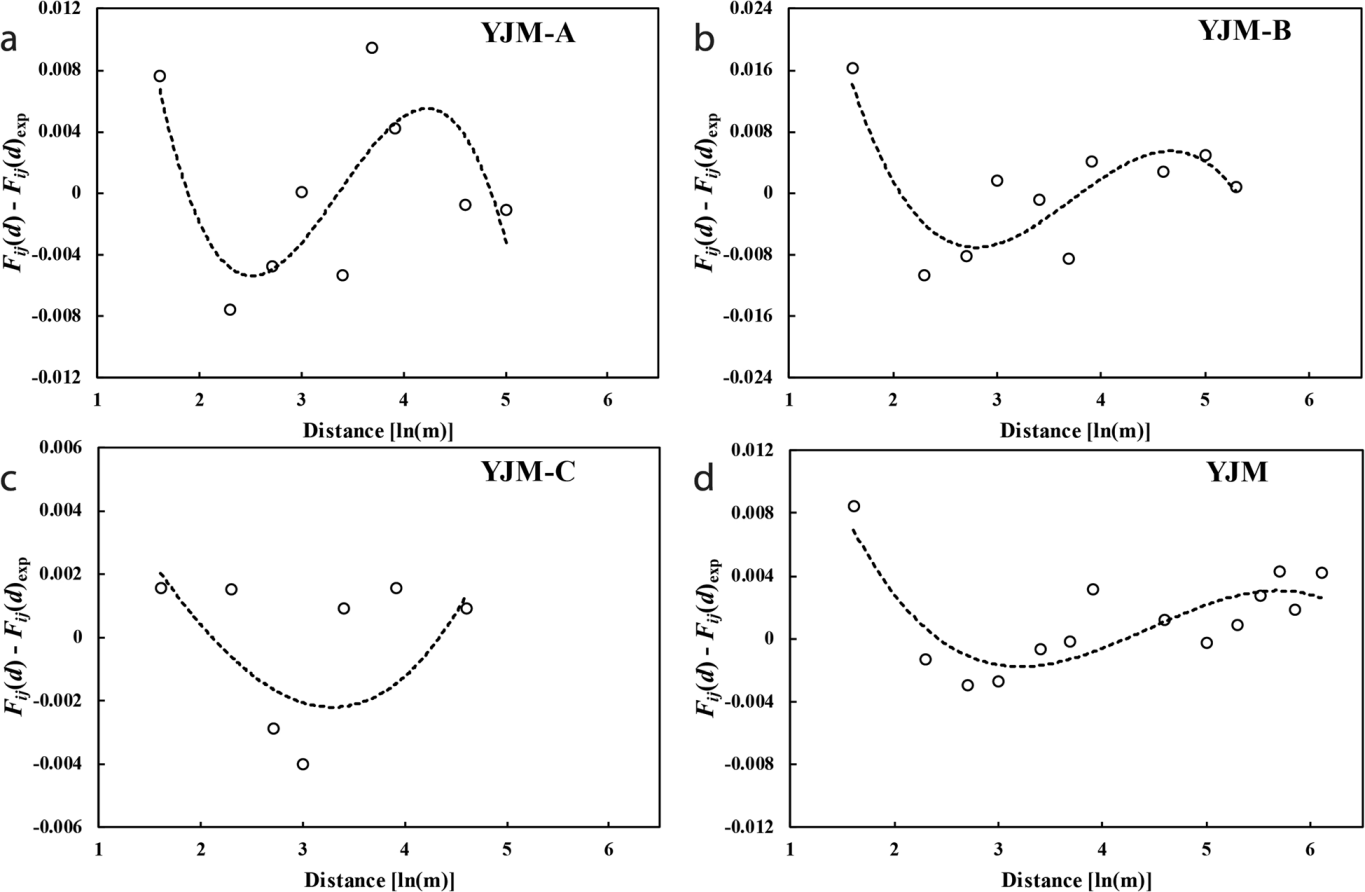
Polynomial regression curves of the third power of residuals [*F*_*ij*_(*d*) - *F*_*ij*_(*d*)_exp_, open circle] on ln(*d*) from sub-population **a** YJM-A, **b** YJM-B, **c** YJM-C and **d** the whole population YJM. All curves are concave at short log distances, indicating that seed dispersal is more restricted than pollen dispersal

Polynomial regression curves of the third power of residuals [*F*_*ij*_(*d*) - *F*_*ij*_(*d*)_exp_] on ln(*d*) for each sub-population and YJM as a whole are shown in Fig. 5. All regression lines are concave at short distances, with the second derivative *k* = 0.0606 at YJM-A, 0.0629 at YJM-B, 0.0012 at YJM-C and 0.0130 for YJM as a whole, indicating that seed dispersal is more restricted than pollen dispersal (σ_s_ ≪ σ_p_).

### Mating system

Multilocus outcrossing rates (*t*_*m*_) for all individual mother plants were equal to 1.000 (Table 4), strongly suggesting that all seedlings were products of outcrosses. Biparental inbreeding rates (*t*_*m*_ - *t*_*s*_) ranged from − 0.284 to 0.130, with a mean (±SE) value of − 0.011 ± 0.047 among F1 seedlings from the eight capsules (Table 4). The *t*_*m*_ - *t*_*s*_ values of the F1 seedlings from capsule IC6, IC7 and AMP3 were less than 0, indicating biparental outbreeding, whereas *t*_*m*_ - *t*_*s*_ values of the F1 seedlings from capsule IC5, HC5, HC3, AMP1 and AMP8 were greater than 0, indicating biparental inbreeding (Table 4).

**Table 4:** Mating system parameters for progeny contained in eight open-pollinated capsules of *Phalaenopsis pulcherrima*. Multilocus outcrossing rate (*t*_*m*_), single-locus outcrossing rate (*t*_*s*_), outcrossing rate among relatives (*t*_*m*_ - *t*_*s*_), calculated from 1000 bootstrap replicates by resampling maternal families (SE)

Maternal plant	No. of F1 Seedlings	*t*_*s*_ (SE)	*t*_*m*_ (SE)	*t*_*m*_*-t*_*s*_ (SE)
IC5	189	0.919 (0.016)	1.000 (0.001)	0.081 (0.016)
IC6	182	1.284 (0.010)	1.000 (0.001)	−0.284 (0.010)
IC7	187	1.052 (0.011)	1.000 (0.001)	−0.052 (0.011)
HC5	56	0.900 (0.016)	1.000 (0.001)	0.100 (0.016)
HC3	181	0.870 (0.040)	1.000 (0.001)	0.130 (0.040)
AMP1	169	0.980 (0.010)	1.000 (0.001)	0.020 (0.010)
AMP8	24	0.995 (0.009)	1.000 (0.001)	0.005 (0.009)
AMP3	112	1.089 (0.013)	1.000 (0.001)	−0.089 (0.013)
Mean		1.011 (0.047)	1.000 (0.001)	−0.011 (0.047)

Analysis of correlated mating patterns revealed significant correlation in outcrossing rates among siblings (*M* ± *SE*, *r*_*t*_ = − 0.999 ± 0.000). Multilocus correlated paternity (*r*_*pm*_, the probability that siblings shared the same father) was 0.999 ± 0.063, and the average effective number of pollen donors per maternal plant (*N*_*ep*_) was equal to one, strongly suggesting that all F1 seedlings derived from a single capsule shared the same pollen parent (Table 5). Comparisons between single- and multi-locus estimates of correlated paternity was equal to 0, indicating that correlated paternity does not occur via related male parents (Table 5).

**Table 5:** Population-level mating system parameters for *Phalaenopsis pulcherrima*. Calculated from 1000 bootstrap replicates by resampling maternal families (SE)

Parameters	Estimates	SE
Correlation of selfing (*r*_*t*_)	−0.999	0.000
Multilocus correlated paternity (*r*_*pm*_)	0.999	0.063
Single locus correlated paternity (*r*_*ps*_)	0.999	0.075
Extent of outcrossed paternity by related male parents (*r*_*ps*_*-r*_*pm*_)	0.000	0.058
Average effective number of pollen donors per maternal plant (*N*_*ep*_)	1.000	–
Fixation index for maternal genotypes (*F*_*m*_)	−0.199	0.090

### Paternity analysis

Analysis of the 1100 F1 seedlings (i.e. excluding the one seedling from capsule CC1 and four seedlings from capsule HC2) assigned 556 (50.5%) and 623 (56.6%) to a single pollen parent in the population at the 95 and 80% confidence levels, respectively. Seedlings assigned to a single pollen parent with 95% confidence were derived from four capsules: AMP1 and AMP3 from YJM-B, and IC6 and HC5 from YJM-C (Table 6). The pollen donor and mother of each of these four capsules belonged to separate sub-populations. All F1 seedlings from a single capsule were full-sibs, indicating one pollen donor per capsule. Pollen dispersal distances for the capsules with confirmed pollen parents ranged from 113.6 m to 345.6 m, with a mean (±SD) distance of 272.7 ± 108.4 m. The remaining 477 F1 seedlings (43.4%) at < 80% confidence levels were not assigned to a pollen parent within the study population, and so were assumed to have been sired by an individual in the population that had not been sampled, or to be the product of immigrant pollen from outside YJM.

**Table 6:** Paternity analysis of 1100 F1 seedlings form eight maternal plants with known maternal genotypes at 95% confidence level. No available information (—)

Maternal plants	No. of F1 seedlings for paternity analysis	Main pollen donors with 95% strict confidence			Pollen dispersal distance
		Paternal plants	No. of F1 seedlings assigned	Assigned proportion	
IC5	189	–	–	–	–
IC6	182	G6	182	100.00	113.6 m
IC7	187	–	–	–	–
HC3	56	–	–	–	–
HC5	181	DC4	181	100.00	294.2 m
AMP1	169	CC2	170	100.00	337.3 m
AMP3	24	A3	24	100.00	345.6 m
AMP8	112	–	–	–	–
Total	1100	4	556	50.5	272.7 m

## Discussion

### Post-pollination floral development and inbreeding depression

Pollination is known to stimulate ovary and ovule development in preparation for fertilization and embryogenesis [40], bringing about a series of changes in flower pigmentation, senescence or abscission of floral organs [41]. Such post-pollination floral development stems from initial pollen-stigma interactions [40, 42] and might represent an evolutionarily stable means of avoiding subsequent pollination events and so reduce pollen wastage [43]. Emasculation in many orchids is known to bring about a similar outcome [e.g. [44]. In this study, the lifespan of an emasculated flower was shown to be significantly shorter than that of a bagged flower. We documented a hitherto unknown mechanism for stigma obfuscation, involving enlargement of the rostellum until it coalesces with and eventually entirely obscures the stigma within a few hours of pollination. This differs from analogous mechanisms described in other orchids, in which the sepals and petals wilt and enclose the column [45], implicating the evolution of more specialized post-pollination floral development in *P. pulcherrima*.

Self-pollination barriers are generally considered rare in orchids [28, 46], but have been confirmed in a growing number of large and ecologically diverse tropical genera, including *Bulbophyllum*, *Dendrobium* and *Oncidium* [27, 46, 47]. In our study, epifluorescence microscopy revealed that pollen grains germinate 3 days after pollination regardless of the source of the pollinia, but that self-pollinated flowers have a lower rate of pollen germination and pollen tube growth than cross-pollinated ones, resulting in low fertilization with a mean seed-set of just 16.1%. These attributes may be indicative of either partial self-incompatibility, most likely due to a late-acting barrier [48], or inbreeding depression, both of which can cause selfed ovules to abort [49]. Whereas embryo abortion is likely to occur more-or-less simultaneously among selfed ovules in late-acting self-incompatibility, in inbreeding depression it can occur at several stages, giving rise to greater variation in seed set among capsules [50]. Since seed-set in selfed capsules ranged from 0.0–72.0% in our experiments, inbreeding depression may be a more plausible explanation in *P. pulcherrima*.

### Genetic diversity and differentiation

Low genetic diversity is usually attributed to a suite of demographic factors, such as a scattered population structure comprising a few small sub-populations, typically compounded by the genetic effects of bottlenecks, inbreeding, fragmentation, limited gene flow and drift [3, 4, 9]. Our study revealed high genetic diversity within populations and low genetic differentiation among sub-populations of *P. pulcherrima*, which may be at least partly attributed to the species’ deceptive pollination system, thereby reducing geitonogamous pollination [17, 18]. Given the observation that selfed capsules in *P. pulcherrima* have a low seed-set, the fact that only five of the 124 open-pollinated capsules monitored in the present study contained seeds with a significantly depressed seed-set suggests that most open-pollinated capsules are probably the result of outcrossing.

In vitro germination remains challenging for many orchid species [51], but the germination rates and resulting genetic data obtained in the present study suggest that recruitment is high and that there is no loss of genetic diversity from one generation to the next. High genetic variation (in terms of both *H*_*o*_ and *H*_*e*_) was confirmed among adult plants, and although our estimates for the F1 seedlings were derived from individuals cultured from only ten capsules, we found no significant difference in genetic diversity among them and the background population in terms of *N*_*a*_, *N*_*e*_, *I*, *H*_*o*_ or *H*_*e*_. Our study also confirmed a low fixation index (*F*_*is*_) within the population and between generations, providing further evidence of a predominantly outcrossing mating system.

### Linking gene flow and spatial ecology

To a large extent, population genetic structuring is determined by seed dispersal distances, regardless of whether pollen dispersal is limited or not [5]. Despite orchid seeds having the potential for long-distance dispersal [30], most experimental data demonstrate the spatial extent of seed-mediated gene flow in orchids to be rather limited [33, 52]. In part, this may be attributed to the exalbuminous orchid seed, which relies on fungal colonization for germination and seedling establishment [53]. Thus, several studies found most recruitment to occur close to mother plants [34–36], sometimes within only a few meters, suggesting declining abundance of mycorrhizal partners in microsites further from adult plants [7, 52]. This can cause significant fine-scale genetic structuring [12, 54, 55]. Our analyses of *P. pulcherrima* revealed both a clumped spatial structure and significant fine-scale genetic structuring, both at the level of the three sub-populations and for YJM as a whole. Moreover, our gene flow dispersal estimates reveal that seed dispersal is more restricted than pollen dispersal, suggesting that the concentration of related individuals at shorter distances (< 10 m) is due overwhelmingly to short-range seed dispersal.

The *Sp* statistic allows patterns of spatial genetic structure across species and studies (even those using different sampling schemes) to be directly compared, despite the fact that higher *Sp* values can indicate stronger genetic structure at smaller spatial scales [37]. Based on data from 47 plant species, Vekemans & Hardy (2004) [5] found the *Sp* statistic to be significantly related to mating system (higher in selfing species), life form (higher in herbs than trees), and population density (higher in more dispersed populations). In comparison to mean values for other species, the *Sp* statistic calculated here for *P. pulcherrima* (0.0030–0.0058) is significantly lower than for both self-pollinating (0.1431) and outcrossing (0.0126) species [5], even when compared with other orchids, e.g. *Orchis purpurea* (0.0144 to 0.0148) [56], *Cyclopogon luteoalbus* (0.053) [57] and *Vanilla humblotii* (0.020 to 0.045) [58]. There are three possible explanations for this [12]. First, our results strongly suggest a predominantly outcrossing mating system in *P. phalaenopsis* i.e. predominance of high seed-set in open-pollinated capsules, a low fixation index and multilocus outcrossing rates. Contrary to selfing species, in which only seed dispersal contributes to overall gene dispersal, pollen dispersal in outcrossing species is likely to reduce genetic differentiation among individuals within populations and thus decrease the overall degree of relatedness and genetic structuring [5]. Second, self-pollinated capsules are predicted to give rise to relatively low seedling recruitment due to the lower viability of selfed ovules as compared to those resulting from outcrossing, and this is likely to reduce genetic structuring and enhance effective population size; conversely, seedling recruitment resulting from outcrossing will lead to a lower proportion of relatives within populations and fewer homozygotic plants. Third, *P. phalaenopsis* commonly grows in open areas on granite outcrops and at the edge of sparse forest [59], habitats that could be exposed to relatively great air movement and therefore conducive to wide seed dispersal. Thus, seeds of *P. phalaenopsis* may disperse relatively far, at least at a local scale, leading to comparatively low intensity fine-scale genetic structuring.

Although a handful of studies have demonstrated that sexual reproduction in some orchids can be brought about by multiple pollen donors acting on a single stigma [13–15], most empirical evidence suggests that the diversity of fathers per capsule is far lower than the total number of available pollen donors [6, 60]. In our study, the average effective number of pollen donors per maternal plant was 1.000, indicating that each of eight open-pollinated capsules were sired by a single pollen donor. In all cases, paternity analysis confirmed the mother plant and pollen donor to be genetically distinct individuals, with a multilocus outcrossing rate of 1.000. We successfully assigned paternity to 556 of 1100 F1 seedlings at the 95% confidence level. Interestingly, the pollen donors for the four capsules which contained these seedlings were situated between 113.6 m to 345.6 m distant from the mother plants, with a mean separation of 272.7 m. In all four cases, the two parent plants were situated in separate sub-populations with intervening dense woodland in which the orchid does not grow, reflecting inferred patterns of pollen immigration among populations of a European orchid [61]. These figures may even underestimate actual pollen dispersal distances, because there remains the possibility that the four capsules for which we were unable to assign paternity were sired by plants outside the study population.

Previous studies that have attempted to estimate pollen flow distances using pollen tracking methods have demonstrated maximum distances in rewarding and deceptive orchids in the range of 7–76 m [62]. To our knowledge, only one other study has assigned paternity using genetic analyses, with pollen dispersal distances in the terrestrial Australian *Chiloglottis* aff. *jeanesii* and *C. valida* found to range from 0 to 69 m with a median value of 14.5 m [15]. The pollen-mediated gene flow distances confirmed in the present study are therefore an order of magnitude greater than those documented elsewhere, and reveal that pollinating bees move across a matrix of habitats, some of which are not suitable for the orchid. However, given that some bees are known to have foraging distances exceeding 10 km, e.g. *Xylocopa virginica* and *Eufriesea surinamensis* [63], it remains plausible that future studies will extend our understanding of long-range pollen flow in orchids even further.

## Conclusions

Here we confirm post-pollination floral development and self-sterility in *Phalaenopsis pulcherrima*. A predominantly outcrossing mating system based on deceptive pollination appears to contribute to high total genetic diversity and low genetic differentiation among sub-populations, as well as significant departure from Hardy-Weinberg equilibrium. The orchid exhibits both a clumped distribution and significant genetic structuring over fine scales, with polynomial regression analysis indicating that seed dispersal is more restricted than pollen dispersal at the scale of the study population. However, *Sp* statistics calculated for *P. pulcherrima* are significantly lower than those for other herbaceous species with similar ecological attributes, possibly owing to the species’ outcrossing mating system, inferred low recruitment from selfed capsules and local seed dispersal. Naturally pollinated capsules each appear to be sired by a single father, with pollen flow distances ranging from 113.6 m to 345.6 m. We detected no loss of genetic diversity from one generation to the next. Taken together, our findings suggest that gene flow in this species is sufficient to maintain high genetic diversity, connectivity among spatially discrete sub-populations and large effective population size.

## Methods

### Study species

*Phalaenopsis pulcherrima* (Lindl.) J.J.Sm. (Orchidaceae) is a diploid (2*n* = 38), perennial, lithophytic or terrestrial herb that is native to most countries of tropical Southeast Asia; in China, it occurs only in Hainan Province [64], where it has declined over the last four decades due to habitat destruction and over-collection for horticulture.

*Phalaenopsis pulcherrima* is capable of both sexual reproduction and asexual clonal propagation [59]. Plants produce up to two erect, racemose inflorescences bearing 5–30 flowers that open acropetally during June–October. Flowers are approximately 2–3 cm across and very variable in color, ranging from pure white to pink or purple. Each flower has four waxy, sub-globose pollinia that are separable from each other but which detach as a unit via a long stipe with a sticky viscidium. *Phalaenopsis pulcherrima* employs a generalized food-deceptive pollination system [59]. In a successful pollination event, the pollinarium adheres to the thorax of a foraging bee, *Amegilla nigritar*, and is transported to a receptive flower [59]. Ramets produced through clonal propagation emerge from a side-shoot within 5 cm of the original plant, with multiple branching producing sizeable clumps comprising many shoots in close proximity.

### Study site

As is typical for the species in other parts of its range [65], in Hainan *P. pulcherrima* grows on exposed granite outcrops or in coarse soils at the edge of thick forest at elevations of 100–800 m [59, 64]. The present study was conducted on a slope of Yiajia mountain (hereafter referred to as YJM) in Bawangling National Nature Reserve (19°7′ N, 109°10′ E), which has a seasonal tropical climate.

**Fig. 6:**
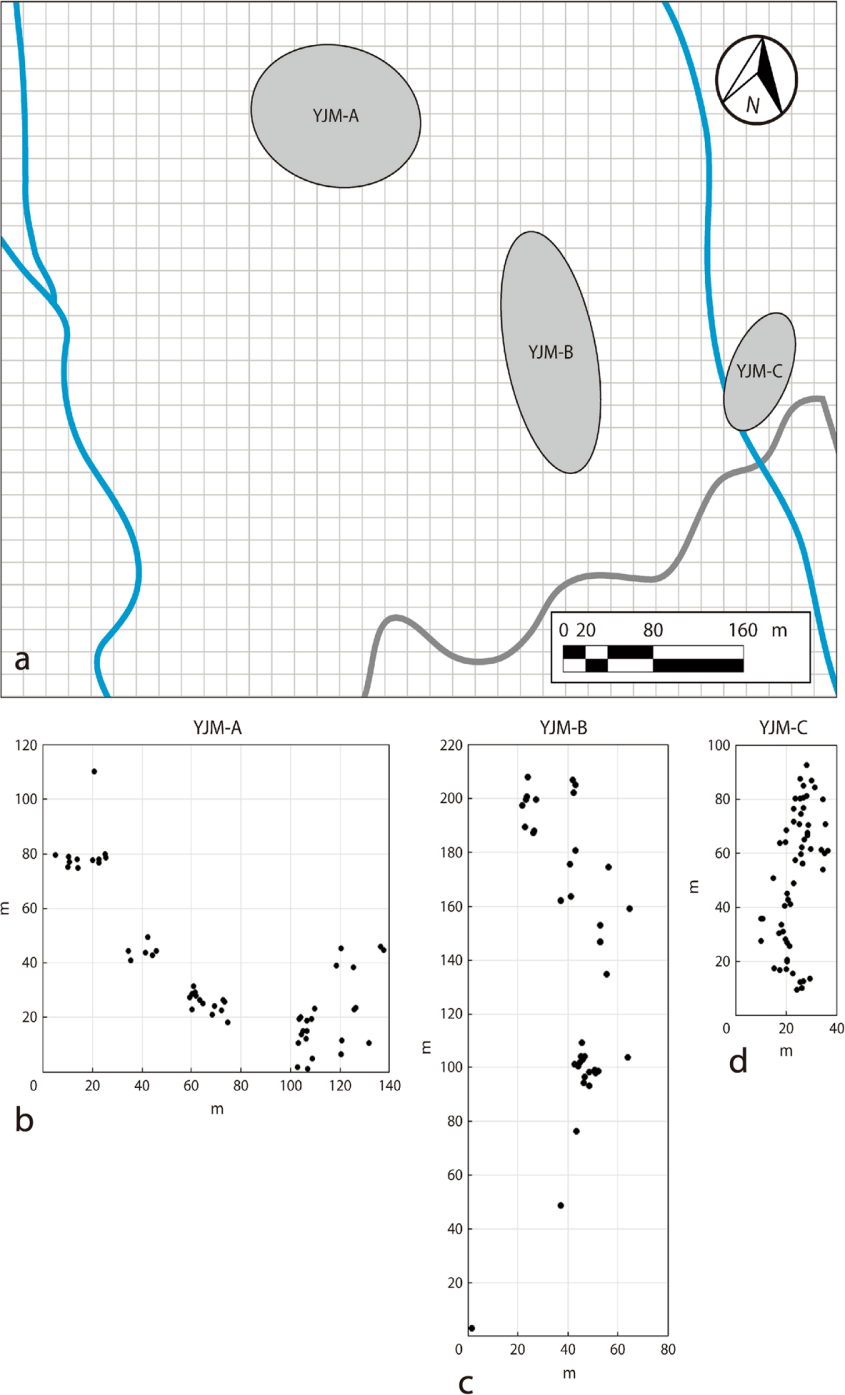
Spatial distribution of the three *Phalaenopsis pulcherrima* sub-populations at (**a**) YJM, and positions of all genets within sub-population (**b**) YJM-A, **c** YJM-B and d) YJM-C. Streams are indicated in blue and a road in grey

Plants at YJM are physically separated into three sub-populations by dense woodland that is unsuitable for the orchid, with intervening distances of > 100 m (Fig. 6); sampling reflected this spatial distribution. The first sub-population (hereafter YJM-A) lies at c. 500 m a.s.l., occupies an area of c. 140 × 120 m and comprises 56 individuals; the second sub-population (YJM-B) lies at c. 300 m a.s.l., occupies an area of c. 80 × 220 m and comprises 39 individuals; the third sub-population (YJM-C) lies at c. 200 m a.s.l., occupies an area of c. 40 × 100 m and comprises 60 individuals. YJM-A and YJM-B occur in partial shade under mixed *Pinus latteri* Mason plantation and secondary scrub, whereas YJM-C occurs in full sun on an exposed rocky shelf beside a stream.

### Sampling

We labeled and mapped all genets isolated by > 10 cm from their nearest neighbor (i.e. twice the distance of normal clonal spread) at each of the three sub-populations; in the case of clumped ramets (i.e. < 10 cm separation), only the central ramet was mapped in order to infer the effect of aggregation through sexual recruitment alone. All 155 individuals at YJM were considered candidate mothers and pollen donors, and leaf samples were immediately placed in silica gel for genotyping. All samples used in this study were collected from YJM and a voucher specimen formally identified by the authors (*Zhang & Song PP2011080811*) has been deposited at HUTB.

The site was revisited later in the season to collect seed capsules. We were able to collect capsules from a total of 51 individuals, but 26 proved to be immature and their seeds were therefore not suitable for micropropagation. Of the remaining 25 capsules, ten yielded seeds capable of germination after surface sterilization with hypochlorous acid and culture for 3 months on a modified Vacin & Went (1949) agar-based medium [66] (Additional file 1: Table S2). Once they had produced roots, the F1 seedlings were transferred to a sterile growth medium and maintained at 25 °C under constant light for up to 3 months. Upon reaching 1.5–2 cm in height, all 1105 F1 seedlings were harvested for genotyping; the seedlings derived from capsules CC1 and HC2 were excluded from mating system and paternity analyses due to low numbers (one and four seedlings, respectively; Additional file 1: Table S1). All field experiments and plant material collection complied with institutional, national and international restrictions and guidelines, and prior permission was obtained from Bawangling National Nature Reserve Administration.

### DNA extraction and SSR genotyping

Total genomic DNA was extracted from leaf tissue using a modified CTAB protocol [67]. Microsatellite markers were newly developed for *P. pulcherrima* using a DNA library enrichment method with magnetic beads. A total of 20 microsatellite markers were tested on four samples from the study site (Gale, Li, Zhang & Fischer, unpublished); 15 of these were found to be polymorphic and to consistently produce distinct allelic signals across all individuals (Additional file 1: Table S3). These markers were therefore applied to all 1260 samples (155 adults plus 1105 seedlings).

PCR amplification of primer pairs was performed with a Veriti 96-Well Thermal Cycler (Applied Biosystems, Foster City, CA, USA) using a 25 μl reaction mix containing 50 ng of DNA template, 1× buffer, MgCl_2_ (2 mM), dNTPs (0.2 mM), two primers (0.2 mM of each) and Pfu DNA polymerase (0.5 U; Aidlab, Beijing, China). Forward primers were labeled with a fluorescent dye (FAM, TAMRA, HEX or ROX; Additional file 1: Table S3). PCR amplifications were performed as follows: an initial denaturation step at 94 °C for 5 min, 35 to 40 cycles at 94 °C for 15 s, annealing at 45 to 54 °C for 15 s, and 72 °C for 20 s, with a final extension at 72 °C for 10 min (annealing temperatures shown in Additional file 1: Table S3). PCR products were resolved on an ABI3730xl Genetic Analyzer (Applied Biosystems, Foster City, CA, USA) with an internal LIZ (500) size standard. Fragment data were analyzed using GENEMARKER ver. 2.4.0 (Softgenetics LLC, State College, PA, USA).

### Flower development and breeding system

To evaluate breeding system, we randomly bagged 111 inflorescences to exclude pollinators and assigned flower buds on each inflorescence to one of four treatments: (1) no pre-treatment to test for spontaneous autogamy; (2) emasculation to test for agamospermy; (3) artificial self-pollination to test for self-compatibility and inbreeding depression; or (4) artificial cross-pollination (using the pollinarium from another individual growing more than 10 m away) to evaluate outbreeding depression. Only one treatment involving two flowers was applied per inflorescence. In parallel, we marked another 556 inflorescences and recorded the total number of flowers and fruit capsules produced to derive an estimate of natural fruit set. All resulting hand- and open-pollinated capsules were harvested after 4 months, and the dust-like seeds they contained were transferred to Eppendorf tubes. Seed-set (the proportion of seeds containing an embryo) was assessed by scoring approximately 100 seeds from each capsule under a light microscope (Olympus BX51 microscope, Tokyo, Japan). A Mann-Whitney U test was performed to compare fruit set and one-way ANOVA with a Tukey test was performed to compare the seed set in SPSS 22.0 (IBM Corp.); all proportions were arcsine square-root transformed prior to analysis.

We also randomly selected ten flowers in each treatment to observe floral development. We monitored the artificially self- and cross-pollinated flowers every hour for changes in gynostemium structure following pollination. To estimate normal floral lifespan, we monitored open-pollinated flowers every day until all perianth parts wilted.

### Epifluorescence microscopy

To test for the presence of a self-pollination barrier, we randomly bagged 30 inflorescences to exclude pollinators, selected two flower buds per inflorescence and then performed one self-pollination and one cross-pollination on either as they opened. The hand-pollinated flowers were collected 1, 2, 3, 4, and 5 days after pollination, and the columns were excised, fixed and stained following Cisneros-López et al. (2010) [68], before being visualized under a UV filter on a Leica DM6000B microscope (Leica Microsystems Inc., Wetzlar, Germany).

### Spatial distribution of adult individuals

We used the non-cumulatively univariate O-ring statistic, O(*r*) [69], to summarize spatial occurrence in each sub-population and at YJM as a whole. O(*r*) was calculated from counts of individuals in concentric circles of radius (*r*), with the maximal ring width set to half the length of the shortest plot width. The analysis was performed with a starting ring width of 1 m and with a 1 m lag distance up to 60 m for YJM-A, up to 40 m for YJM-B, up to 20 m for YJM-C and up to 160 m for all individuals at YJM as a whole. The 95% confidence envelopes (CI) about the null hypothesis of complete spatial randomness were constructed from the 25th lowest and 25th highest values computed from 999 replicates by Monte Carlo simulation. The observed spatial distribution was classified as aggregated, random or regular depending on whether the value for O(*r*) was located above, within or below the confidence envelopes [69]. First-order intensity (λ), which indicates average intensity of the point pattern, was also calculated. All calculations and simulations were performed using PROGRAMITA [69]. In addition, we regressed the slope [*b*_LO(*r*)_, the linear regression of O(*r*) on ln(*r*)] and estimated the 95% CI to test whether the slope differed significantly from the null hypothesis [when *b*_LO(*r*)_ = 0]. Calculated slopes for each sub-population were considered to differ significantly if their 95% CIs did not overlap.

### Fine-scale genetic structure and dispersal estimates

To quantify the scale of genetic structuring, we conducted spatial autocorrelation analysis by calculating the pairwise kinship coefficient between individuals (*F*_*ij*_) [10, 70], within sub-populations and at YJM as a whole. To visualize genetic structuring, we calculated mean *F*_*ij*_ for each distance interval, *d*, and plotted this against distance. To meet these conditions, we calculated the mean *F*_*ij*_ (*d*) estimates for intervals defined as 0–20 m (*d* = 5 m), 21–50 m (*d* = 10 m), 51–350 m (*d* = 50 m) and 351–450 m (end-point). Thus, we recognized 14 intervals up to 150 m at YJM-A, 11 intervals up to 200 m at YJM-B, eight intervals up to 100 m at YJM-C, and 14 intervals up to 450 m at YJM. 95% confidence intervals (CI) associated with the null hypothesis of no genetic structure [*F*_*ij*_ (*d*) = 0] were constructed by using 1000 random permutations. *F*_*ij*_ (*d*) was considered to indicate significantly positive or negative scale genetic structure at distance *d* if the 95% CIs did not overlap.

To test whether the slope differed significantly from the null hypothesis of no genetic structure [when *b*_LF(*d*)_ = 0], we regressed the slope [*b*_LF(*d*)_: the linear regression of *F*_*ij*_ (*d*) on ln(*d*)] and estimated the 95% CI by performing 1000 random permutations. The values of *b*_LF(*d*)_ were used to compare the differences among sub-populations by constructing 95% CIs which were obtained as ±1.96 times the SE estimates derived from jackknifing. Slopes for each sub-population were considered to differ significantly if their 95% CIs did not overlap. To compare the overall intensity of fine-scale genetic structure among sub-populations, we also calculated the *Sp* statistic [5], given by *Sp* = − *b*_LF(*d*)_ /[1 − F (1)], where F(1) is the average kinship coefficient between individuals of the first distance class *F*_*ij*_ (5 m).

We also estimated the relative contribution of pollen (σ_p_) and seed (σ_s_) dispersal to total gene flow (σ) within sub-populations and YJM as a whole [71, 72]. Using the average *F*_*ij*_ (*d*) for total samples at each sub-population, we regressed the residuals [*f*(*d*): *F*_*ij*_ (*d*)- *F*_*ij*_ (*d*)_exp_] on ln(*d*) by a polynomial regression of the third power: *f*(*d*) = a + b ln(*d*) + c [ln(*d*)]^2^ + d [ln(*d*)]^3^, where *F*_*ij*_ (*d*)_exp_ is the dependent variable of the linear regression equation at independent variable ln(*d*). The curvature of *f*(*d*) is given by the second derivative, *k* = 2c + 6d ln(*d*_1_), where *d*_1_ is the average distance of the first distance class [e.g. 37]. A concave curve at short distances or *k* > 0 suggests more restricted seed dispersal than pollen dispersal (σ_s_ ≪ σ_p_), whereas a convex shape or *k* < 0 suggests more restricted pollen dispersal or no particular restriction in seed dispersal (σ_s_ ≥ σ_p_) [5]. Statistics were calculated in SPAGEDI [73] and SPSS.

### Genetic diversity and differentiation

The presence of null alleles was checked for using MICRO-CHECKER version 2.2.3 [74] and departure from Hardy–Weinberg equilibrium (HWE) was tested using GENEPOP ver. 4.2 [75]. We tested deviation from the null hypothesis *H*_*0*_ = random union of gametes (*p* < 0.05) and further evaluated the hypothesis when *H*_*1*_ = heterozygote deficiency or *H*_*1*_ = heterozygote excess using a global HWE test [75] with all Markov chain parameters (dememorization, number of batches and number of iterations per batch) set to 10,000. We then calculated the following summary statistics for the 15 microsatellite loci: number of alleles per locus (*N*_*a*_), number of effective alleles (*N*_*e*_), Shannon’s information index (*I*), observed heterozygosity (*H*_*o*_), expected heterozygosity (*H*_*e*_) and inbreeding coefficient (*F*_*is*_) per locus for both maternal plants and F1 seedlings, and a mean value among sub-populations, using GENALEX version 6.502 [76]. The total paternity exclusion probability of the first [Pr(*Ex1*)] and second parent [Pr(*Ex2*)] was calculated using CERVUS 3.0.3 [77, 78]. To test whether genetic diversity differed between adults and F1 seedlings or among sub-populations, one-way ANOVA with a Tukey test was applied for multiple comparisons in SPSS. The estimated value of *F*_*is*_ was also compared to zero. We determined the level of genetic differentiation among the three sub-populations using *F*_*st*_ [79] and standardized genetic differentiation *G’*_*st*_ [80] using AMOVA in GENALEX.

### Mating system and paternity analysis

Estimates of mean multilocus (*t*_*m*_) and single locus (*t*_*s*_) outcrossing rates, correlation of *t*_*m*_ within progeny arrays (*r*_*t*_), multilocus correlated paternity (*r*_*pm*_), single locus correlated paternity (*r*_*ps*_) and fixation index for maternal genotypes (*F*_*m*_) were calculated using MLTR win 3.4 [81], which is based on the multilocus mixed-mating model and assumes progeny are derived from either random mating (outcrossing) or self-fertilization [82]. Biparental inbreeding was estimated following Ritland (1990) [83] as *t*_*m*_ - *t*_*s*_, extent of outcrossed paternity by related male parents was estimated as *r*_*ps*_ - *r*_*pm*_, and the effective number of pollen donors across all mother plants was estimated under the sibling pair model [84] by the relative effective number of pollen donors *N*_*ep*_ = 1/*r*_*pm*_. The program was run using default values for the outcrossing rate (*t* = 0.9), parental inbreeding (*F* = 0.1) and paternity correlation (*r*_*p*_ = 0.1). The estimation of mating system indices was made by the expectation–maximization method to ensure convergence; 1000 bootstraps were used to calculate standard error.

Paternity analysis was conducted in CERVUS, which uses a likelihood-based approach to assign paternity according to the highest logarithm of likelihood (LOD) score. LOD scores were calculated by determining the likelihood of assignment of a parent relative to the likelihood of arbitrary parents. We applied the following simulation parameters to find the confidence level of paternity analysis assignment: 10,000 simulated mating events; 310 candidate paternal plants; 0.50 as the proportion of candidate parents sampled; 0.9941 as the proportion of loci typed; 0.0120 as the rate of typing error; 95% for the strict confidence level; and 80% for the relaxed confidence level.

## Supplementary information

**Additional file 1: Table S1.** Fruit-set in *Phalaenopsis pulcherrima* following artificial pollination treatments (self- and cross-pollination) and natural (open) pollination **Table S2.** Number of F1 seedlings for micropropagation, **Table S3.** Characterization and annealing temperatures (*T*_a_) of 15 microsatellite loci developed for *Phalaenopsis pulcherrima*, .

**Additional file 2: Figure S1.** Pollen grain germination and pollen tube growth in *Phalaenopsis pulcherrima* on successive days after artificial self- and cross-pollination treatment. (a), self-pollination + 2 d, no pollen grain germination; (b), cross-pollination + 2 d, no pollen grain germination; (c), self-pollination + 3 d, extensive pollen grain germination and initial pollen tube growth; (d), cross-pollination + 3 d, pollen tubes extending into style to depth of > 100 μm; (e), self-pollination + 4 d, pollen tubes extending into style to depth of < 200 μm; (f), cross-pollination + 4 d, pollen tubes extending to base of style to depth of 500 μm; (g), self-pollination + 5 d, most pollen tube growth arrested in style, few tubes have penetrated the ovary and contacted ovules. (h), cross-pollination + 5 d, extensive pollen tube growth into ovary with many contacting ovules. P: pollinia, PT: pollen tube, St: stigma, Ov: ovary, Oc: oocyte, and Zy: zygote. Scale bars: a, b) = 250 μm; c, d, e, f) = 100 μm; (g, h) = 50 μm.

## Data Availability

The datasets used and analysed during the current study are available from the corresponding author on reasonable request.

## Abbreviations

*b*_LF(*d*)_: The slope of the regression of F_*ij*_ (*d*) against ln(*d*)
*b*_LO(*r*)_: The slope of the linear regression of the O(*r*) against ln(*r*)
*F*_*ij*_: Kinship coefficients
*F*_*is*_: Inbreeding coefficient
*F*_*m*_: Fixation index for maternal genotypes
*F*_*st*_: F-statistics
*H*_*e*_: Expected heterozygosity
*H*_*o*_: Observed heterozygosity
HWE: Hardy–Weinberg equilibrium
*I*: Shannon’s information index
Km: Kilometer
ln(*d*): Log interval distance
ln(*r*): log spatial distance
*N*: Number of individuals
*N*_*a*_: No. of observed alleles
*N*_*e*_: No. of effective alleles
*N*_*ep*_: Average effective number of pollen donors per maternal plant
O(*r*): O-ring statistic
Pr (*Ex1*): Paternity exclusion probability of the first parent
Pr (*Ex2*): Paternity exclusion probability of the second parent
*r*_*pm*_: Multilocus correlated paternity
*r*_*ps*_: Single locus correlated paternity
*r*_*ps*_-*r*_*pm*_: Extent of outcrossed paternity by related male parents
*r*_*t*_: Correlation of selfing
SE: Standard error
*T*_a_: Annealing temperature
*t*_*m*_ - *t*_*s*_: Outcrossing rate among relatives
*t*_*m*_: Multilocus outcrossing rate
*t*_*s*_: Single-locus outcrossing rate
