# Supplementary material for: Pollen-mediated gene flow ensures connectivity among spatially discrete sub-populations of Phalaenopsis pulcherrima, a tropical food-deceptive orchid
Source: BMC Plant Biol. 2019 Dec 30;19:597. doi: 10.1186/s12870-019-2179-y (PMC6937714; doi:10.1186/s12870-019-2179-y)
Supplement: Supplementary file 1 — Additional file 1: Table S1. Fruit-set in Phalaenopsis pulcherrima following artificial pollination treatments (self- and cross-pollination) and natural (open) pollination Table S2. Number of F1 seedlings for micropropagation, Table S3. Characterization and annealing temperatures (Ta) of 15 microsatellite loci developed for Phalaenopsis pulcherrima, . [file 12870_2019_2179_MOESM1_ESM.docx]

**Table S1** Fruit-set in *Phalaenopsis pulcherrima* following artificial pollination treatments (self- and cross-pollination) and natural (open) pollination.

| Treatments | No. of inflorescences | No. of flowers | No. of fruits | % fruit set |
| --- | --- | --- | --- | --- |
| Bagged only | 10 | 10 | 0 | 0 |
| Emasculation | 10 | 10 | 0 | 0 |
| Self-pollination | 42 | 83 | 74 | 89.16 |
| Cross-pollination | 46 | 94 | 85 | 90.43 |
| Open pollination | 556 | 3243 | 124 | 3.82 |

**Table S2** Number of F1 seedlings for micropropagation

| Sub-population | Maternal plant | No. of F1 Seedlings |
| --- | --- | --- |
| YJM-A | CC1 | 1 |
| YJM-B | AMP1 | 169 |
|  | AMP8 | 24 |
|  | AMP3 | 112 |
| YJM-C | HC2 | 4 |
|  | IC5 | 189 |
|  | IC6 | 182 |
|  | IC7 | 187 |
|  | HC5 | 56 |
|  | HC3 | 181 |
| Total | 10 | 1105 |

**Table S3** Characterization and annealing temperatures (*T*_a_) of 15 microsatellite loci developed for *Phalaenopsis pulcherrima*.

| Locus | Motif | Allele range (bp) | *T*_a_(℃) | Labels |
| --- | --- | --- | --- | --- |
| L3 | (AG)_10_ | 160-220 | 50 | 5'-HEX |
| L6 | (CT)_7_(CA)_16_ | 170-220 | 50 | 5'-TAMRA |
| L9 | (TA)_14_ | 290-340 | 50 | 5'-ROX |
| L22 | (TA)_14_ | 110-170 | 53 | 5'-FAM |
| L29 | (AG)_12_ | 160-220 | 50 | 5'-TAMRA |
| L31 | (AC)_14_(AT)_8_ | 120-180 | 48 | 5'-HEX |
| L33 | (AC)_15_ | 240-290 | 50 | 5'-ROX |
| L46 | (GT)_20_ | 350-370 | 45 | 5'-HEX |
| L51 | (TC)_16_(TC)_19_ | 160-230 | 55 | 5'-ROX |
| L52 | (TC)_13_ | 130-150 | 50 | 5'-FAM |
| L53 | (CT)_12_(TC)_5_ | 180-200 | 52 | 5'-FAM |
| L54 | (CT)_18_ | 140-200 | 52 | 5'-TAMRA |
| L56 | (TC)_13_ | 210-230 | 53 | 5'-ROX |
| L57 | (TC)_13_(TC)_6_ | 120-180 | 52 | 5'-HEX |
| L64 | (CT)_14_ | 160-220 | 55 | 5'-ROX |
