# Supplementary figures and images for: Pollen-mediated gene flow ensures connectivity among spatially discrete sub-populations of Phalaenopsis pulcherrima, a tropical food-deceptive orchid
Source: BMC Plant Biol. 2019 Dec 30;19:597. doi: 10.1186/s12870-019-2179-y (PMC6937714; doi:10.1186/s12870-019-2179-y)

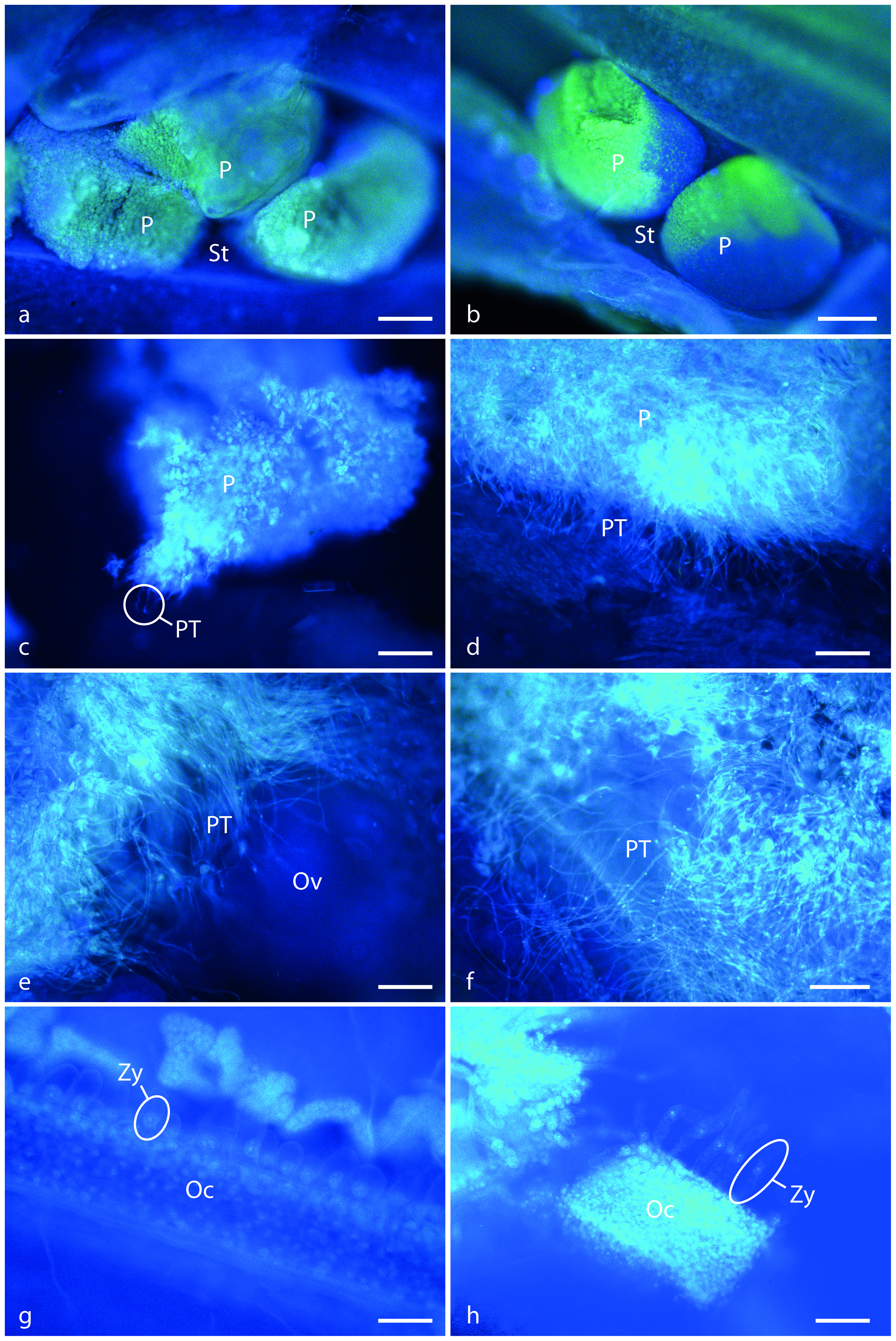

Supplement: Supplementary file 2 — Additional file 2: Figure S1. Pollen grain germination and pollen tube growth in Phalaenopsis pulcherrima on successive days after artificial self- and cross-pollination treatment. (a), self-pollination + 2 d, no pollen grain germination; (b), cross-pollination + 2 d, no pollen grain germination; (c), self-pollination + 3 d, extensive pollen grain germination and initial pollen tube growth; (d), cross-pollination + 3 d, pollen tubes extending into style to depth of > 100 μm; (e), self-pollination + 4 d, pollen tubes extending into style to depth of < 200 μm; (f), cross-pollination + 4 d, pollen tubes extending to base of style to depth of 500 μm; (g), self-pollination + 5 d, most pollen tube growth arrested in style, few tubes have penetrated the ovary and contacted ovules. (h), cross-pollination + 5 d, extensive pollen tube growth into ovary with many contacting ovules. P: pollinia, PT: pollen tube, St: stigma, Ov: ovary, Oc: oocyte, and Zy: zygote. Scale bars: a, b) = 250 μm; c, d, e, f) = 100 μm; (g, h) = 50 μm. [file 12870_2019_2179_MOESM2_ESM.jpg]
